# Supplementary material for: Safety Evaluation by Phenotypic and Genomic Characterization of Four Lactobacilli Strains with Probiotic Properties
Source: Microorganisms. 2022 Nov 9;10(11):2218. doi: 10.3390/microorganisms10112218 (PMC9696993; doi:10.3390/microorganisms10112218)
Supplement: Supplementary file 1 [file microorganisms-10-02218-s001.zip › microorganisms-1953918-supplementary.pdf]

Supplementary Materials

Table S1. Target antibiotics for search in the genome of four *Lactobacillus* species.

No.	Antibiotics	Class
1	Vancomycin	Glycopeptide
2	Ampicillin	Beta-lactam
3	Clindamycin	Lincosamide
4	Erythromycin	Macrolide
5	Kanamycin	Aminoglycoside
6	Gentamicin	Aminoglycoside
7	Chloramphenicol	Phenicol
8	Tetracycline	Tetracycline
9	Streptomycin	Aminoglycoside

Table S2. Summary statistics of the virulence factor database (VFDB) used for search in the genome of four *Lactobacillus* species.

Type of data*	Number
Bacterial pathogens (by genus)	74
of which with full information	32
Virulence factors	1381
of which experimentally verified	1173
Bacteria strains involved	954
of which with complete genome	532
VF-related genes (non-redundant)	32827
of which with curation	3580
Related literatures	3197

*Current dataset (http://www.mgc.ac.cn/VFs/)

Table S3. Biogenic production of four *Lactobacillus* species.

Strain	Biogenic amine (mM)					
	**Tyramine**	**Histamine**	**Putrescine**	**2-Phenethylamine**	**Cadaverine**	**Tryptamine**
L. fermentum IDCC 3901	n.d.[1]	n.d.	n.d.	n.d.	n.d.	n.d.
L. gasseri IDCC 3101	n.d.	n.d.	n.d.	n.d.	n.d.	n.d.
L. helveticus IDCC 3801	n.d.	n.d.	n.d.	n.d.	n.d.	n.d.
L. salivarius IDCC 3551	n.d.	n.d.	n.d.	n.d.	n.d.	n.d.

[1]n.d. not detected

Table S4. L- and D-lactate production by four *Lactobacillus* species.

Strain	L–lactate (g/L)	D–lactate (g/L)	Ratio	
			**L-lactate**	**D-lactate**
L. fermentum IDCC 3901	14.33 ± 0.21	7.98 ± 0.18	64.23	35.7
L. gasseri IDCC 3101	16.52 ± 0.44	7.56 ± 0.25	68.6	31.4
L. helveticus IDCC 3801	24.83 ± 0.15	13.98 ± 0.27	63.9	36.1
L. salivarius IDCC 3551	28.12 ± 0.15	3.24 ± 0.03	89.6	10.4

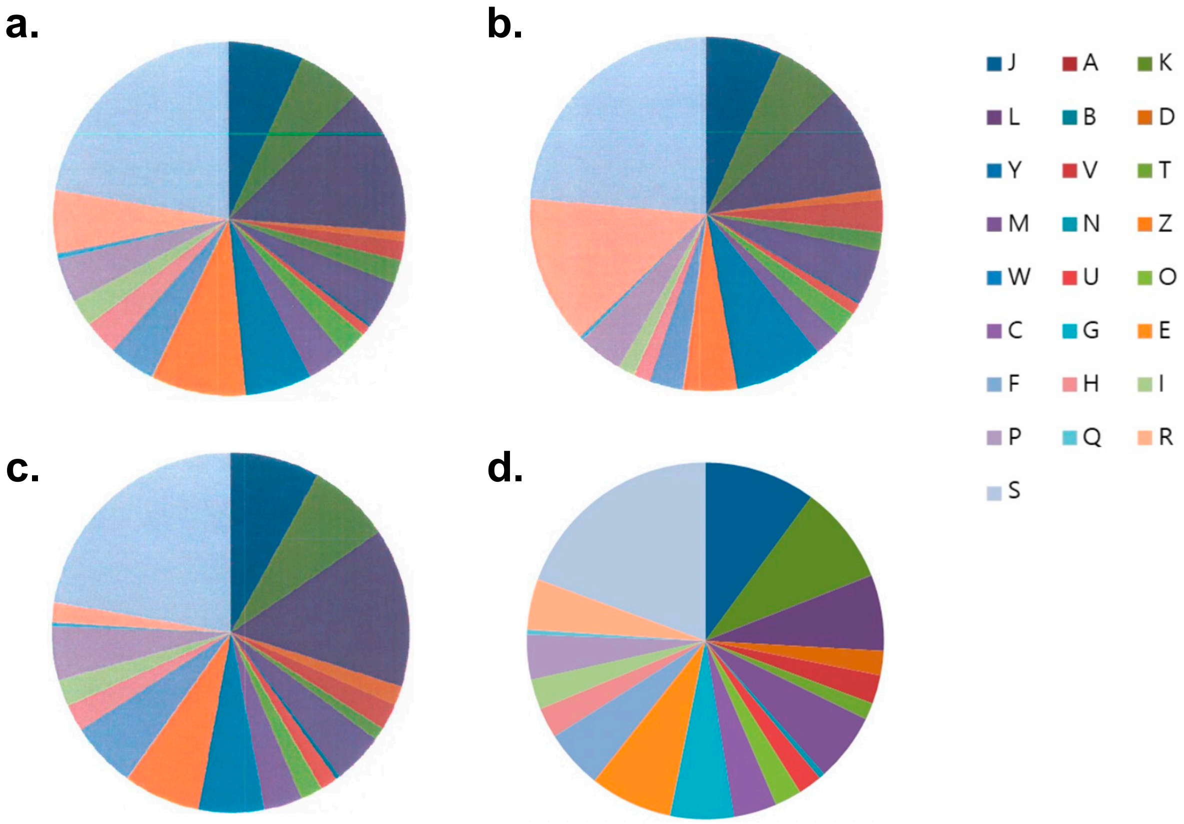

Figure S1. Functional genes of four *Lactobacillus* species.
Functional genes were annotated and grouped using the eggnog database. J, translation, ribosomal structure and biogenesis; A, RNA processing and modification; K, transcription; L, replication, recombination, and repair; D, cell cycle control, cell division, chromosome partitioning; Y, nuclear structure; V, defense mechanisms; T, signal transduction mechanisms; M, cell wall/membrane/envelope biogenesis; N, cell motility; Z, cytoskeleton; W, extracellular structures; U, intracellular trafficking, secretion, and vesicular transport; O, posttranslational modification, protein turnover, and chaperones; C, energy production and conversion; G, carbohydrate transport and metabolism; E, amino acid transport and metabolism; F, nucleotide transport and metabolism; H, coenzyme transport and metabolism; I, lipid transport and metabolism; P, inorganic ion transport and metabolism; Q, secondary metabolite biosynthesis, transport, and catabolism; R, general function prediction only; S, function unknown.
